# Impact of Healthy Lifestyle in Patients With Familial Hypercholesterolemia

**DOI:** 10.1016/j.jacasi.2022.10.012

**Published:** 2023-01-31

**Authors:** Hayato Tada, Nobuko Kojima, Kan Yamagami, Akihiro Nomura, Atsushi Nohara, Soichiro Usui, Kenji Sakata, Kenshi Hayashi, Noboru Fujino, Masayuki Takamura, Masa-aki Kawashiri

**Affiliations:** a Department of Cardiovascular Medicine, Graduate School of Medical Sciences, Kanazawa University, Kanazawa, Japan; b Department of Clinical Genetics, Ishikawa Prefectural Central Hospital, Kanazawa, Japan; c Department of Internal Medicine, Kaga Medical Center, Kaga, Japan

**Keywords:** familial hypercholesterolemia, genetics, LDL cholesterol, LDL receptor, lifestyle, CVD, cardiovascular disease, FH, familial hypercholesterolemia, LDL, low-density lipoprotein, MACE, major adverse cardiac event(s)

## Abstract

**Background:**

Pathogenic mutations are associated with poor outcomes in patients with familial hypercholesterolemia (FH). However, data on the effects of a healthy lifestyle on FH phenotypes are limited.

**Objectives:**

The authors investigated the interaction between a healthy lifestyle and FH mutation with prognosis in patients with FH.

**Methods:**

We investigated the associations of the interaction between genotypes and lifestyle, with the occurrence of major adverse cardiac events (MACE), such as cardiovascular-related mortality, myocardial infarction, unstable angina, and coronary artery revascularization, in patients with FH. We assessed their lifestyle based on 4 questionnaires (healthy dietary pattern, regular exercise, not smoking, and absence of obesity). The Cox proportional hazards model was used to assess the risk for MACE.

**Results:**

The median follow-up duration was 12.6 (IQR: 9.5-17.9) years. During the follow-up duration, 179 MACE were observed. Independent of classic risk factors, FH mutation and lifestyle score were significantly associated with MACE (HR: 2.73; 95% CI: 1.03-4.43; *P =* 0.02; and HR: 0.69, 95% CI: 0.40-0.98, *P =* 0.033, respectively). The estimated risk of coronary artery disease by 75 years of age varied according to lifestyle, ranging from 21.0% among noncarriers with a favorable lifestyle to 32.1% among noncarriers with an unfavorable lifestyle and ranging from 29.0% among carriers with a favorable lifestyle to 55.4% among carriers with an unfavorable lifestyle.

**Conclusions:**

A healthy lifestyle was associated with reduced risk for MACE among patients with FH with or without genetic diagnosis.

Familial hypercholesterolemia (FH) is caused by a pathogenic mutation in the low-density lipoprotein (LDL) receptor (*LDLR*) or its associated genes, including apolipoprotein B (*APOB*), proprotein convertase subtilisin/kexin type 9 (*PCSK9*), and *LDLR* adaptor protein 1 (*LDLRAP1*).^1^ Patients with FH have an extremely elevated risk for cardiovascular disease (CVD) due to high LDL cholesterol levels, beginning at birth. Although the prevalence of this disease is 1 in 300 in the general population, the prevalence increases to 1 in 15 patients with early onset CVD.^2^^,^^3^ Furthermore, this disease is inherited; thus, pathogenic mutations such as FH convey an increased CVD risk.^4^^,^^5^ Other acquired factors, including hypertension, diabetes, and smoking, are considered additional risk factors for FH.^6^^,^^7^

Studies have shown that a healthy lifestyle reduced the risk of CVD among the general population^8^^,^^9^ and individuals with increased inherited risk attributed to common genetic variations.^10^ However, data on the effect of a healthy lifestyle on FH phenotypes caused by a rare genetic variation are limited. Despite their high genetic risk for CVD, it is important to clarify if a healthy lifestyle can mitigate an extremely elevated risk in these individuals. We set up a hypothesis that a healthy lifestyle is associated with reduced risk for CVD among patients with FH. Therefore, we investigated the interaction between a healthy lifestyle and FH mutation with prognosis in patients with FH.

## Methods

### Study population

We assessed the data of 2,011 patients with FH diagnosed clinically using the 2017 Japan Atherosclerosis Society criteria^11^ at Kanazawa University Hospital between 1990 and 2020. Briefly, all patients fulfilled at least 2 of the 3 essential clinical criteria stipulated by the Japan Atherosclerosis Society for FH diagnosis. The criteria are as follows: 1) LDL cholesterol level of ≥180 mg/dL; 2) tendon xanthoma on the back of the hands, elbows, knees, or other areas; Achilles tendon hypertrophy or Achilles tendon thickness (≥9 mm) on radiography; or xanthoma tuberosum; and 3) family history of FH or premature coronary artery disease diagnosed in a first- or second-degree relative. In total, 536 patients were excluded due to missing data, 6 for genetic variations, and 508 were lost to follow-up. Finally, 961 patients were included in this study (Supplemental Figure 1).

### Clinical data assessment

Hypertension was defined as a systolic blood pressure of ≥140 mm Hg, diastolic blood pressure of ≥90 mm Hg, or the use of antihypertensive medications. We used the definition of diabetes established by the Japan Diabetes Society.^12^ Smoking status was defined as current smoking. CVD was defined as the presence of angina pectoris, myocardial infarction, or severe stenotic region(s) in the coronary artery (≥75% stenosis) on angiography or computed tomography. The serum total cholesterol, triglyceride, and high-density lipoprotein cholesterol levels were determined enzymatically using automated instrumentation. The LDL cholesterol level was calculated using the Friedewald formula if the patient’s triglyceride level was <400 mg/dL. Otherwise, it was evaluated enzymatically. Major adverse cardiac events (MACE) were defined as mortality associated with CVD, myocardial infarction, unstable angina, or staged revascularization. LDL cholesterol year score was calculated as: LDL cholesterol max × (age at diagnosis/statin initiation) + LDL cholesterol at inclusion × (age at inclusion − age at diagnosis/statin initiation).

### Genetic analysis

Genotypes were examined using a next-generation sequencer. In brief, the coding regions of the *LDLR*, *APOB, PCSK9*, and *LDLRAP1* were sequenced, as described in a previous study.^13^ Furthermore, copy number variations at the *LDLR* were assessed using the eXome Hidden Markov Model, as previously described.^14^ Finally, the pathogenicity of genetic variants was evaluated using the standard American College of Medical Genetics and Genomics criteria.^15^

### Ascertainment of lifestyle risk score

We used data from questionnaires administered during routine health checkups at the inclusion. In Japan, a uniform questionnaire regarding healthy lifestyle factors has been used since 2008.^16^ We made a 4-point score for the lifestyle assessment, including healthy dietary patterns, regular exercise, not smoking, and absence of obesity, as previously described.^17^ One point was given if the patient’s lifestyle fulfilled the following criteria. The first was a healthy dietary pattern. Patients were given 1 point if they did not answer “yes” to any of the following questions: 1) eating faster than people of the same generation; 2) eating dinner within 2 hours before going to bed (more than 3 times a week); 3) having a snack after dinner (more than 3 times a week); and 4) skipping breakfast more than 3 times a week. The second was regular exercise: patients were given 1 point if they had a habit of exercising (>30 minutes, twice a week, >1 year). The third was not smoking: patients were given 1 point if they did not have current smoking status. The fourth was absence of obesity: patients were given 1 point if their body mass index was <25.0 kg/m^2^. We classified patients into 3 healthy lifestyle categories: unfavorable (score = 0-2), intermediate (score = 3), and favorable (score = 4).

### Ethical considerations

This study was approved by the Ethics Committee of Kanazawa University. All procedures were conducted following the ethical standards of the Human Research Committee (institutional and national) and the 1975 Declaration of Helsinki (revised in 2008). Informed consent for genetic analysis was obtained from all participants.

### Statistical analysis

Continuous variables with a normal distribution are expressed as mean ± SD. Continuous variables with a non-normal distribution are presented as median (IQR). Categorical variables are reported as number and percentage and compared using Fisher exact or chi-square tests. The mean values of continuous variables were compared using Student’s *t*-test for independent variables, and the median values were compared using the Mann-Whitney *U* test. The chi-square or Fisher exact test was used for categorical variables as appropriate. The Cox proportional hazards model was used to assess associations between variables. In addition, HRs were calculated in several different models, such as: 1) lifestyle characteristics (healthy dietary pattern, regular exercise, not smoking, and absence of obesity); 2) each lifestyle score (unfavorable, intermediate, and favorable); and 3) 6 subgroups divided by 2 carrier statuses × 3 lifestyle score categories. We quantified the age-dependent probability of MACE in carriers of an FH mutation and in noncarriers of different lifestyle scores. We fit a Cox proportional hazards regression model with age as the time scale, defining the time to event as the age at which the diagnosis was first ascertained among patients with MACE and the age at the most recent follow-up among those without MACE. The probability of disease by time t was estimated by F(t) = 1 − S(t), where S(t) is the survivor function, calculated by the survfit function from the R survival package, version 4.1.0 (R Foundation for Statistical Computing). All statistical analyses were conducted using R. *P* values of <0.05 were considered statistically significant.

## Results

### Clinical characteristics of the participants

Table 1 shows the participants’ clinical characteristics. The patients’ mean age was 52 years, and almost half were men. The median LDL cholesterol level at baseline was 234 mg/dL. Furthermore, 294 (30.6%) patients presented with a history of CVD. A total of 699 patients had an FH mutation (Supplemental Table 1).

**Table 1:** Baseline Characteristics

	All (N = 961)	FH Mutation (+) (n = 699)	FH Mutation (−) (n = 262)	*P* Value
Age, y	52 ± 16	50 ± 16	56 ± 15	3.90 × 10^-7^
Male	449 (46.7)	328 (46.9)	121 (46.2)	0.8946
Body mass index, kg/m^2^	23.0 ± 0.6	22.8 ± 0.6	23.3 ± 0.6	0.18
Hypertension	276 (28.7)	213 (30.5)	63 (24.0)	0.06
Diabetes	83 (8.6)	69 (9.9)	14 (5.3)	0.036
Total cholesterol, mg/dL	319 (287-360)	323 (290-372)	304 (278-334)	3.40 × 10^-8^
Triglycerides, mg/dL	125 (84-173)	122 (78-168)	142 (105-178)	4.70 × 10^-5^
HDL cholesterol, mg/dL	47 (40-57)	46 (39-56)	49 (41-59)	0.02334
LDL cholesterol at baseline, mg/dL	234 (206-279)	251 (216-292)	209 (196-229)	<2.20 × 10^-16^
LDL cholesterol at follow-up, mg/dL	110 (93-131)	117 (99-137)	93 (82-110)	<2.20 × 10^-16^
History of prior CVD	294 (30.6)	238 (34.0)	56 (21.4)	0.0002
Lifestyle characteristics				
Healthy dietary pattern	740 (77.0)	527 (75.4)	213 (81.3)	0.06419
Regular exercise	451 (46.9)	311 (44.5)	140 (53.4)	0.01634
Not smoking	660 (68.7)	491 (70.2)	169 (64.5)	0.1031
Absence of obesity	864 (89.9)	632 (90.4)	232 (88.5)	0.4626
Lifestyle score	2.8 ± 0.9	2.8 ± 0.9	2.9 ± 0.9	0.2533
Favorable	243 (25.3)	176 (25.2)	67 (25.5)	0.9667
Intermediate	374 (38.9)	262 (37.5)	112 (42.7)	0.1566
Unfavorable	344 (35.8)	261 (37.3)	83 (31.7)	0.1201

Values are mean ± SD, n (%), or median (IQR).

CVD = cardiovascular disease; FH = familial hypercholesterolemia; HDL = high-density lipoprotein; LDL = low-density lipoprotein.

Compared with noncarriers, FH mutation carriers were younger and were more likely to have diabetes and a prior history of CVD. FH mutation carriers additionally exhibited higher levels of total and LDL cholesterol. There was no significant difference in lifestyle characteristics except for the percentage of patients with regular exercise habits. We identified 86 pathogenic mutations in 699 patients.

We described the characteristics of the patients excluded in this study due to loss of follow-up (Supplemental Table 2). We observed significant differences in several variables including age, hypertension, diabetes, total cholesterol, triglycerides, LDL cholesterol, prior CVD, healthy dietary pattern, and nonsmoking.

### Factors associated with MACE

During the median follow-up duration of 12.6 years, 179 patients experienced MACE, which included CVD-associated mortality, myocardial infarction, unstable angina, and staged revascularization (Table 2). Using the Cox proportional hazards model, we assessed factors correlated with MACE. Results showed that age (adjusted HR: 1.07; 95% CI: 1.04-1.10; *P =* 2.40 × 10^-11^), male sex (adjusted HR: 1.67; 95% CI: 1.10-2.24; *P =* 0.006), hypertension (adjusted HR: 2.54; 95% CI: 1.88-3.20; *P =* 6.90 × 10^-5^), diabetes (adjusted HR: 1.87; 95% CI: 1.24-2.50; *P =* 0.003), LDL cholesterol year score (adjusted HR, per 1,000 mg-y/dL: 1.29; 95% CI: 1.03-1.55; *P =* 0.0021), previous history of CVD (adjusted HR: 3.12; 95% CI: 2.00-4.24; *P* < 2.2 × 10^-16^), FH mutation (adjusted HR: 2.73; 95% CI: 1.03-4.43; *P =* 0.02), and lifestyle score (adjusted HR: 0.69; 95% CI: 0.40-0.98; *P =* 0.033) were significantly associated with MACE (Table 3).

**Table 2:** Major Adverse Cardiac Events (N = 961)

Death associated with CVD	56 (5.8)
Myocardial infarction	24 (2.5)
Unstable angina	33 (3.4)
Staged revascularization	67 (6.7)
Total	179 (18.6)

Values are n (%).

CVD = cardiovascular disease.

**Table 3:** Factors Associated With MACE

	HR	95% CI	*P* Value
Age (per year)	1.07	1.04-1.10	2.40 × 10^-11^
Male (yes vs no)	1.67	1.10-2.24	0.006
Hypertension (yes vs no)	2.54	1.88-3.20	6.90 × 10^-5^
Diabetes (yes vs no)	1.87	1.24-2.50	0.003
LDL cholesterol (per 10 mg/dL, at baseline)	1.00	1.00-1.00	0.08
LDL cholesterol year score (per 1,000 mg-y/dL)	1.29	1.03-1.55	0.0021
Prior CVD (yes vs no)	3.12	2.00-4.24	<2.20 × 10^-16^
FH mutation (yes vs no)	2.73	1.03-4.43	0.02
Lifestyle score (per 1 point)	0.69	0.40-0.98	0.033

**Figure 1:**
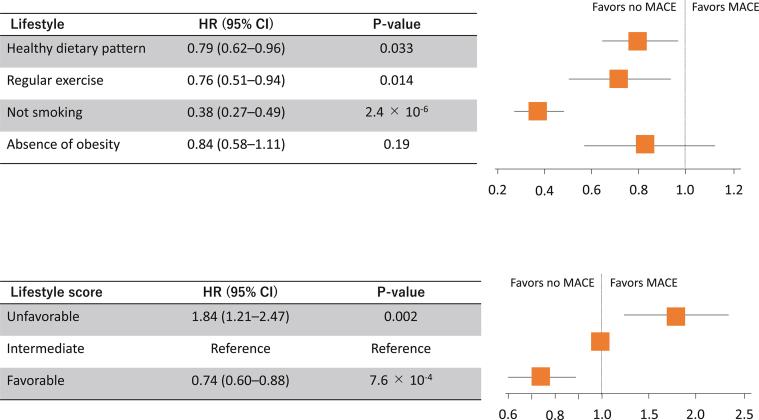
Healthy Lifestyle and Risk for MACE **(Top)** HRs adjusting for age, sex, hypertension, diabetes, low-density lipoprotein cholesterol, and history of prior cardiovascular disease for major adverse cardiac events (MACE) of each healthy lifestyle factor are illustrated. **(Bottom)** HRs adjusting for age, sex, hypertension, diabetes, low-density lipoprotein cholesterol, and history of prior cardiovascular disease for MACE of each healthy lifestyle factor are illustrated.

MACE = major adverse cardiac event(s); other abbreviations as in Figure 1.

### Healthy lifestyle and risk for MACE

Among the 4 healthy lifestyle factors, healthy dietary pattern, regular exercise, and not smoking were significantly associated with a decreased risk of MACE (adjusted HR: 0.79; 95% CI: 0.62-0.96; *P =* 0.033; adjusted HR: 0.76; 95% CI: 0.51-0.94; *P =* 0.014; and adjusted HR: 0.38; 95% CI: 0.27-0.49; *P =* 2.40 × 10^-6^, respectively), while absence of obesity was not significantly associated with MACE (adjusted HR: 0.84; 95% CI, 0.58-1.11; *P =* 0.19) (Figure 1). Compared with those with an intermediate lifestyle (score of 3), the HR for the risk of MACE for those with an unfavorable lifestyle (score of 0-2) was 1.84 (95% CI, 1.21-2.47; *P =* 0.002), and that for the risk of MACE for those with a favorable lifestyle (score of 4) was 0.74 (95% CI, 0.60-0.88; *P =* 7.60 × 10^-4^) (Figure 1).

### Risk of MACE among FH mutation carriers and noncarriers according to lifestyle

**Figure 2:**
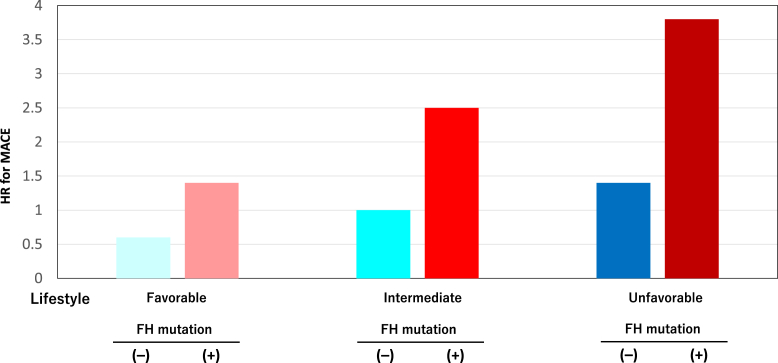
Risk of MACE According to Lifestyle The y-axis represents the HR for major adverse cardiac events (MACE). HR was calculated after adjusting for age, sex, hypertension, diabetes, low-density lipoprotein cholesterol, and history of prior cardiovascular disease. **Light blue** indicates patients without pathogenic mutations and a favorable lifestyle. **Blue** indicates patients without pathogenic mutations and an intermediate lifestyle. **Dark blue** indicates patients without pathogenic mutations and an unfavorable lifestyle. **Light red** indicates patients with pathogenic mutations and a favorable lifestyle. **Red** indicates patients with pathogenic mutations and an intermediate lifestyle. **Dark red** indicates patients with pathogenic mutations and an unfavorable lifestyle. FH = familial hypercholesterolemia.

Compared with noncarriers with an intermediate lifestyle, carriers with a favorable lifestyle had an adjusted HR of 1.4 (95% CI: 0.6-3.6) (*P =* 0.44) (Figure 2), while carriers with an unfavorable lifestyle had an adjusted HR of 3.8 (95% CI: 2.0-5.6) (*P =* 2.40 × 10^-4^) (Figure 2).

### Age-dependent probability of CVD according to FH mutation and lifestyle

**Figure 3:**
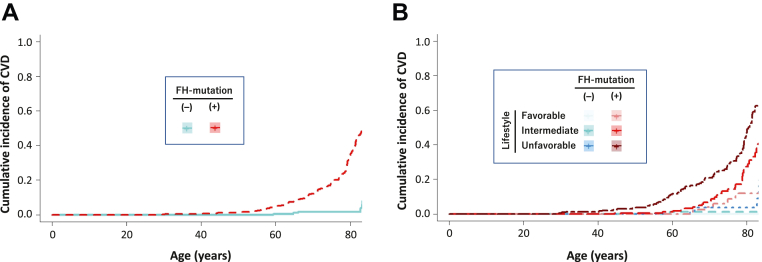
Age-Dependent Probability of CVD According to FH Mutation and Lifestyle We quantified the age-dependent probability of cardiovascular disease (CVD) in carriers of a familial hypercholesterolemia (FH) mutation and in noncarriers of different lifestyle scores. **(A) Red** indicates patients with pathogenic mutations. **Blue** represents patients without pathogenic mutations. **(B) Light blue** indicates patients without pathogenic mutations and a favorable lifestyle. **Blue** indicates patients without pathogenic mutations and an intermediate lifestyle. **Dark blue** indicates patients without pathogenic mutations and an unfavorable lifestyle. **Light red** indicates patients with pathogenic mutations and a favorable lifestyle. **Red** indicates patients with pathogenic mutations and an intermediate lifestyle. **Dark red** indicates patients with pathogenic mutations and an unfavorable lifestyle.

**Figure 4:**
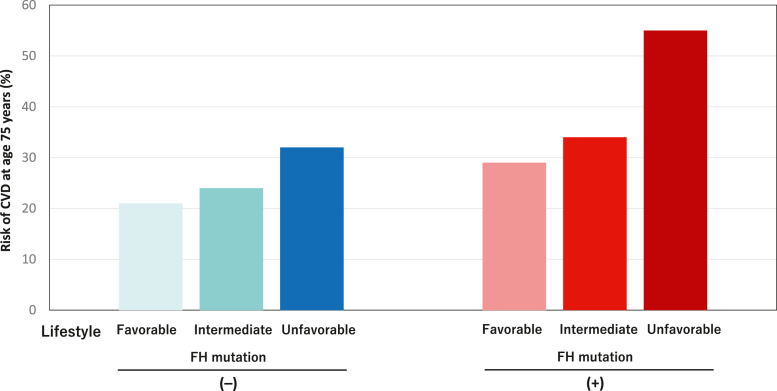
Probability of CVD at the 75 Years of Age According to FH Mutation and Lifestyle The y-axis represents the risk of CVD at 75 years of age. HR was calculated after adjusting for age, sex, hypertension, diabetes, low-density lipoprotein cholesterol, and history of prior CVD. **Light blue** indicates patients without pathogenic mutations and a favorable lifestyle. **Blue** indicates patients without pathogenic mutations and an intermediate lifestyle. **Dark blue** indicates patients without pathogenic mutations and an unfavorable lifestyle. **Light red** indicates patients with pathogenic mutations and a favorable lifestyle. **Red** indicates patients with pathogenic mutations and an intermediate lifestyle. **Dark red** indicates patients with pathogenic mutations and an unfavorable lifestyle. Abbreviations as in Figure 3.

FH mutation carriers exhibited earlier incidence of CVD compared with noncarriers (Figure 3A). Moreover, we see earlier incidence of CVD among patients with a worse genetic status and/or worse lifestyle score category when we divided patients into 6 groups based on 2 carrier status × 3 lifestyle score categories (Figure 3B). Using a Cox proportional hazards regression model, we then estimated the age-dependent probability of manifesting MACE across a strata of FH mutations and lifestyle characteristics, noting a gradient of risk by 75 years of age, ranging from 21.0% for those with no FH mutation and a favorable lifestyle to 32.1% among those with no FH mutation and an unfavorable lifestyle and from 29.0% among those with an FH mutation and a favorable lifestyle to 55.4% for those with both an FH mutation and an unfavorable lifestyle (Figure 4).

## Discussion

We evaluated the effects of FH mutation and a healthy lifestyle on the clinical phenotypes of FH. Patients with an FH mutation had elevated risk than noncarriers. Notably, a healthy lifestyle was associated with reduced risk for CVD regardless of FH mutation status (Central Illustration).

**Central Illustration:**
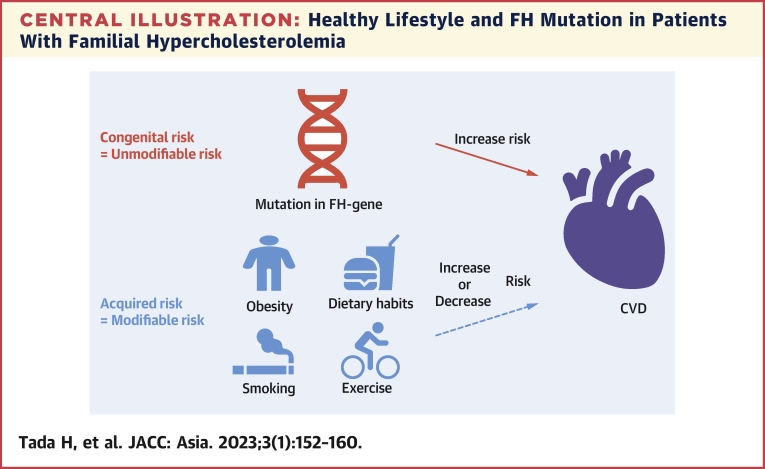
Healthy Lifestyle and FH Mutation in Patients With Familial Hypercholesterolemia A mutation in familial hypercholesterolemia (FH) gene illustrated in **red** is considered to be a congenital (unmodifiable) risk for cardiovascular disease (CVD). On the other hand, a healthy lifestyle illustrated in **blue** is considered to be an acquired (modifiable) risk for CVD that can ease their innate high risk for CVD.

Patients with FH have an extremely high CVD risk based on their elevated LDL cholesterol levels, beginning at birth.^18^ LDL-lowering therapies should be considered as early as possible for these patients.^19^, ^20^, ^21^ In addition to medications, adherence to a healthy lifestyle has been recommended as the first step in FH clinical guidelines.^11^ In fact, a healthy lifestyle is associated with reduced risk for CVD among general populations; however, there are few data regarding this matter among patients with FH. Fahed et al^17^ showed that adherence to a healthy lifestyle pattern was associated with reduced risk for CVD regardless of FH mutation status, suggesting that patients with FH are likely to benefit from lifestyle interventions to reduce their risk of CVD.

In this study, we have verified that this concept applies to patients with clinically diagnosed FH regardless of FH mutation status. These results reassure us that clinical and genetic diagnosis of FH is not deterministic of risk for CVD. Rather, we are delighted to find that inherited risk can be mitigated by a healthy lifestyle.

It is sometimes difficult to assess if the patient’s lifestyle is “healthy” because there are different concepts of what constitutes a healthy lifestyle. To this end, the American Heart Association recently proposed the Life’s Simple 7 concept.^22^ This simple score comprises 7 risk factors, including blood pressure, cholesterol, blood sugar, and 4 lifestyle-related factors (physical activity, healthy diet, managing body weight, and not smoking). Life’s Simple 7 is associated with various CVDs^23^^,^^24^ and is easily assessed due to its simplicity. We, and others, use 4 lifestyle-related factors (physical activity, healthy diet, managing body weight, and no smoking) as a simple surrogate marker for “lifestyle.” These 4 lifestyle-related factors appear to adequately reflect if a particular lifestyle is “healthy.” However, we acknowledge that smoking is the most important risk factor in lifestyle. Although smoking affects the development of atherosclerosis in non-FH individuals, it looks like there is an even stronger association between smoking and atherosclerosis in FH, due to the fact that at least a part of the mechanisms of atherogenicity of smoking is the oxidative stress.^25^ In fact, it has been shown that smoking is associated with oxidized LDL cholesterol.^26^ Accordingly, the amount of oxidized LDL cholesterol can be greatly increased when the patients with FH smoke. On the other hand, obesity appears to affect less to the results in this study. This is probably due to the fact that most of the patients with FH in this study were not obese. Thus, further studies including more obese patients are needed to clarify this issue.

In addition to their lifestyle, we observed that LDL cholesterol year score, which reflects lifelong exposure to LDL cholesterol, was significantly associated with MACE. This clearly suggests that they need to be treated earlier on top of lifestyle changes.

### Study limitations

First, the study was retrospective and conducted at a single center. Therefore, the findings might not apply to other patients. However, our institution has a long history of treating patients with FH and has one of the largest databases in Japan. Second, we could not account for treatments administered during follow-up because there were a variety of treatments, which might have affected the study results. Third, several patients were excluded from the analysis due to missing data or were lost to follow-up. This exclusion could have affected the study findings. Fourth, a functional analysis was not performed to validate the pathogenicity of genetic mutations. Hence, some pathogenic and benign mutations were classified as having unknown significance. Fifth, polygenic factors were not considered. Therefore, further studies must be performed to facilitate comprehensive risk assessments. Sixth, we could not account for the changes of their lifestyle during follow-up period, which could affect the results. However, it is almost impossible to account for the changes, if any, of their lifestyle, although many of them are taking annual health checkups. Moreover, other studies have also shown the usefulness of such scores assessed only once.^10^^,^^27^ Seventh, smoking appears to play a major role in this score. In this matter, we and others have shown that smoking is significantly associated with MACE among patients with FH.^5^^,^^28^^,^^29^ So, it should be noted that abstention from smoking is the primary goal for this high-risk group. Nevertheless, much data are needed to clarify which lifestyle factor should be targeted, particularly, among patients with FH. Eighth, we defined an absence of obesity as a healthy lifestyle. However, obesity is a complex multifactorial disease, and may not be a simple surrogate for lifestyle.

## Conclusions

A healthy lifestyle was associated with a reduced risk of CVD among patients with FH regardless of their FH mutation status. Therefore, lifestyle modifications should be considered in patients with FH to mitigate their inherited cardiovascular risk. However, we need to clarify when and which factor in particular should be considered as interventions for lifestyle in this high-risk group in future studies.

Perspectives **COMPETENCY IN MEDICAL KNOWLEDGE:** The present study showed that a healthy lifestyle was associated with reduced risk for CVD regardless of FH mutation status, suggesting that a clinical and genetic diagnosis as FH is not deterministic for risk for CVD.

**TRANSLATIONAL OUTLOOK:** Future studies are needed to clarify when and which factor in particular should be considered as interventions for lifestyle in this high-risk group of patients.

## Funding Support and Author Disclosures

This work was supported by the JSPS KAKENHI (20H03927, 21H03179, and 22H03330). Dr Tada has received a grant from the Ministry of Health, Labor and Welfare of Japan (Sciences Research Grant for Research on Rare and Intractable Diseases) and the Japanese Circulation Society (project for genome analysis in cardiovascular diseases) and the Japan Agency for Medical Research and Development (20314864 and 22672854). All other authors have reported that they have no relationships relevant to the contents of this paper to disclose.
