# Supplementary material for: Impact of Healthy Lifestyle in Patients With Familial Hypercholesterolemia
Source: JACC Asia. 2023 Jan 31;3(1):152–60. doi: 10.1016/j.jacasi.2022.10.012 (PMC9982286; doi:10.1016/j.jacasi.2022.10.012)
Supplement: Supplemental Figure 1 and Tables 1 and 2 [file mmc1.docx]

**Supplemental Figure 1. STROBE flowchart**

**
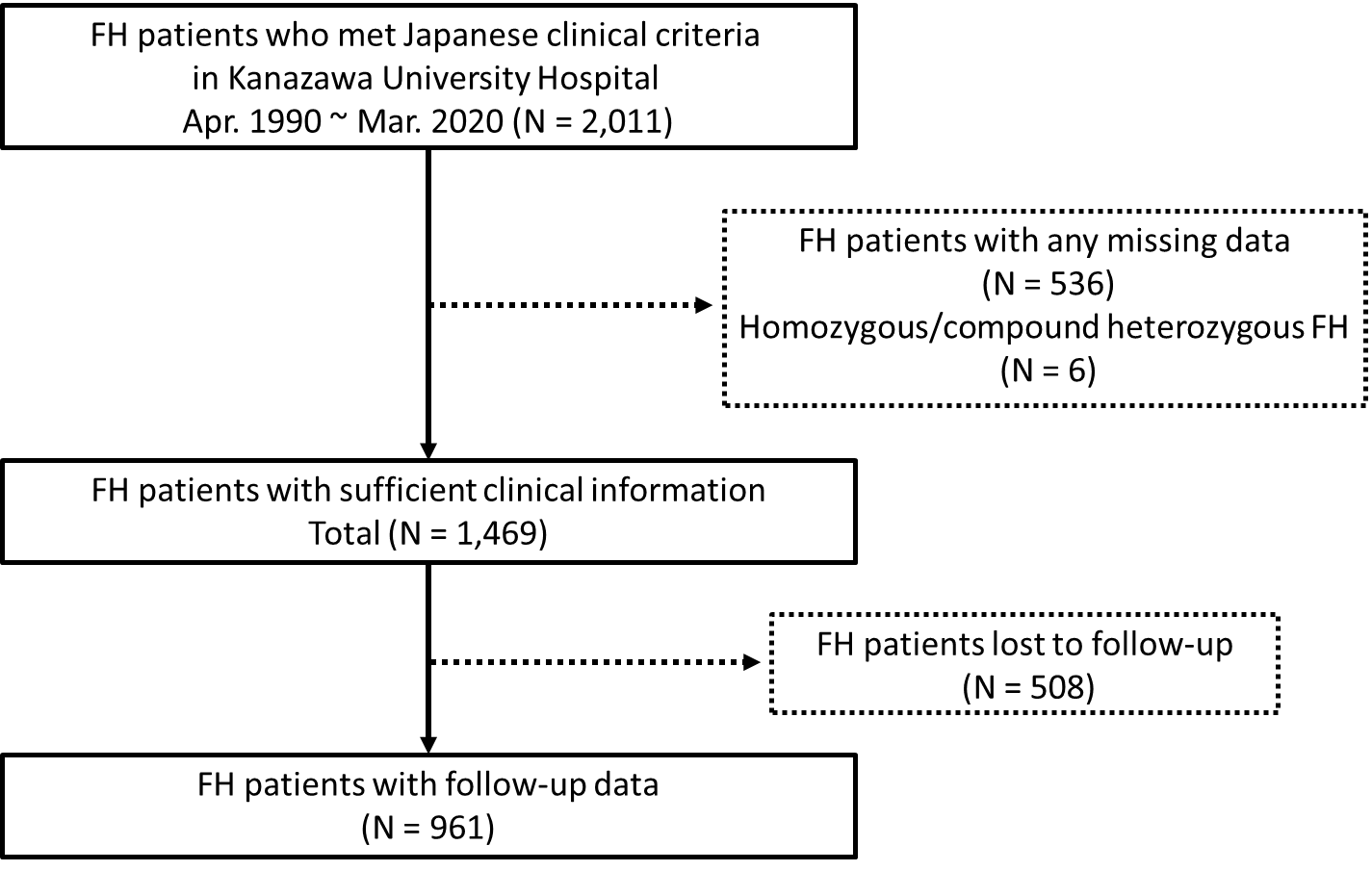
**

**Supplemental Table 1. Pathogenic variants in this study**

| Gene | Nucleotide change | Mutation type | Effect on protein | Number of patients | ACMG |  |
| --- | --- | --- | --- | --- | --- | --- |
| *LDLR* | c.14G>A | Missense | p.Gly5Asp | 4 | PM1/PM2/PP3/PP4 Likely pathogenic |  |
| *LDLR* | c.68-1G>C | Splice-cite | NA | 2 | PVS1/PM2/PM4/PP1/PP5 Pathogenic |  |
| *LDLR* | c.130T>G | Missense | p.Trp44Gly | 3 | PM1/PM2/PP3/PP4 Likely pathogenic |  |
| *LDLR* | c.137G>A | Missense | p.Cys46Tyr | 4 | PM1/PM2/PP3/PP5 Likely pathogenic |  |
| *LDLR* | c.191-2A>G | Splice-cite | NA | 6 | PVS1/PM2/PM4/PP1 Pathogenic |  |
| *LDLR* | c.283T>G | Missense | p.Cys95Gly | 4 | PM1/PM2/PP3/PP4 Likely pathogenic |  |
| *LDLR* | c.284G>T | Missense | p.Cys95Phe | 3 | PM2/PM5/PP1/PP5 Likely pathogenic |  |
| *LDLR* | c.313+1G>A | Splice-cite | NA | 2 | PVS1/PM2/PM4 Pathogenic |  |
| *LDLR* | c.344G>A | Missense | p.Arg115His | 3 | PS1/PM2/PP3 Likely pathogenic |  |
| *LDLR* | c.361T>G | Missense | p.Cys121Gly | 5 | PM1/PM2/PP3/PP4/PP5 Likely pathogenic |  |
| *LDLR* | c.378del | Frameshift | p.Phe126LeufsTer80 | 4 | PVS1/PM2/PM4/PP1 Pathogenic |  |
| *LDLR* | c.389dupC | Frameshift | p.Asp131ArgfsTer49 | 5 | PVS1/PM1/PM2/PM4/PP5 Pathogenic |  |
| *LDLR* | c.413C>G | Nonsense | p.Ser138Ter | 6 | PVS1/PM2/PM4/PP1/PP5 Pathogenic |  |
| *LDLR* | c.418G>A | Missense | p.Glu140Lys | 4 | PM1/PM2/PP3/PP5 Likely pathogenic |  |
| *LDLR* | c.478T>C | Missense | p.Cys160Arg | 4 | PM1/PM2/PP3/PP5 Likely pathogenic |  |
| *LDLR* | c.489G>T | Missense | p.Gln163His | 4 | PM1/PM2/PP3/PP5 Likely pathogenic |  |
| *LDLR* | c.530C>T | Missense | p.Ser177Leu | 4 | PM1/PM2/PP3/PP5 Likely pathogenic |  |
| *LDLR* | c.532G>T | Missense | p.Asp178Tyr | 5 | PM1/PM2/PP3/PP5 Likely pathogenic |  |
| *LDLR* | c.611G>C | Missense | p.Cys204Ser | 6 | PM1/PM2/PP3/PP5 Likely pathogenic |  |
| *LDLR* | c.642G>C | Missense | p.Trp214Cys | 7 | PM1/PM2/PP3/PP5 Likely pathogenic |  |
| *LDLR* | c.682G>A | Missense | p.Glu228Gln | 5 | PM1/PM2/PP3/PP5 Likely pathogenic |  |
| *LDLR* | c.686_689del | Frameshift | p.Glu229AlafsTer35 | 4 | PVS1/PM2/PM4/PP1 Pathogenic |  |
| *LDLR* | c.694+1G>A | Splice-cite | NA | 2 | PVS1/PM2/PM4/PP1 Pathogenic |  |
| *LDLR* | c.718G>A | Missense | p.Glu240Lys | 3 | PM1/PM2/PP3/PP5 Likely pathogenic |  |
| *LDLR* | c.718G>T | Nonsense | p.Glu240Ter | 2 | PM1/PM2/PP3/PP4 Likely pathogenic |  |
| *LDLR* | c.726G>C | Missense | p.Gln242His | 4 | PM1/PM2/PP3/PP4 Likely pathogenic |  |
| *LDLR* | c.796G>A | Missense | p.Asp266Asn | 4 | PM1/PM2/PP3/PP4 Likely pathogenic |  |
| *LDLR* | c.797A>G | Missense | p.Asp266Gly | 11 | PM1/PM2/PM5/PP1/PP3 Likely pathogenic |  |
| *LDLR* | c.829G>T | Nonsense | p.Glu277Ter | 2 | PVS1/PM2/PM4/PP1 Pathogenic |  |
| *LDLR* | c.874delC | Frameshift | p.Leu292TrpfsTer78 | 3 | PVS1/PM2/PM4/PP1 Pathogenic |  |
| *LDLR* | c.901G>T | Missense | p.Asp301Tyr | 5 | PM1/PM2/PP3/PP5 Likely pathogenic |  |
| *LDLR* | c.902A>T | Missense | p.Asp301Val | 4 | PM1/PM2/PP3/PP5 Likely pathogenic |  |
| *LDLR* | c.937T>G | Missense | p.Cys313Gly | 3 | PM1/PM2/PP3/PP4 Likely pathogenic |  |
| *LDLR* | c.939C>A | Missense | p.Cys313Ter | 2 | PVS1/PM2/PM4/PP5 Pathogenic |  |
| *LDLR* | c.940+2T>C | Splice-cite | NA | 2 | PVS1/PM2/PM4/PP1 Pathogenic |  |
| *LDLR* | c.967G>A | Missense | p.Gly323Ser | 4 | PM1/PM2/PP3/PP4 Likely pathogenic |  |
| *LDLR* | c.1007_1010delACGA | Frameshift | p.Tyr336CysfsTer33 | 2 | PVS1/PM2/PM4/PP5 Pathogenic |  |
| *LDLR* | c.1012T>A | Missense | p.Cys338Ser | 2 | PM1/PM2/PP3/PP5 Likely pathogenic |  |
| *LDLR* | c.1056C>A | Nonsense | p.Cys352Ter | 4 | PVS1/PM2/PM4/PP5 Pathogenic |  |
| *LDLR* | c.1062dupT | Frameshift | p.Ile355TyrfsTer3 | 5 | PVS1/PM2/PM4/PP5 Pathogenic |  |
| *LDLR* | c.1067A>T | Missense | p.Asp356Val | 4 | PM1/PM2/PP3/PP4 Likely Pathogenic |  |
| *LDLR* | c.1069G>T | Nonsense | p.Glu357Ter | 2 | PVS1/PM2/PM4 Pathogenic |  |
| *LDLR* | c.1114_1115insC | Frameshift | p.Glu372AlafsTer9 | 4 | PVS1/PM2/PM4/PP5 Pathogenic |  |
| *LDLR* | c.1123_1124insGA | Nonsense | p.Tyr375Ter | 2 | PVS1/PM2/PM4/PP5 Pathogenic |  |
| *LDLR* | c.1183_1184insC | Frameshift | p.Val395AlafsTer46 | 2 | PVS1/PM2/PM4/PP5 Pathogenic |  |
| *LDLR* | c.1187-2A>G | Splice-cite | NA | 3 | PVS1/PM2/PM4/PP5 Pathogenic |  |
| *LDLR* | c.1207T>C | Missense | p.Phe403Leu | 2 | PM1/PM2/PP3/PP5 Likely pathogenic |  |
| *LDLR* | c.1245_1249dupCCGGA | Frameshift | p.Ser417ThrfsTer12 | 4 | PVS1/PM2/PM4/PP4 Pathogenic |  |
| *LDLR* | c.1246C>T | Missense | p.Arg416Trp | 4 | PM1/PM2/PP3/PP4 Likely pathogenic |  |
| *LDLR* | c.1252G>A | Missense | p.Glu418Lys | 6 | PM1/PM2/PP3/PP4 Likely pathogenic |  |
| *LDLR* | c.1285G>A | Missense | p.Val429Leu | 8 | PM1/PM2/PP3/PP4 Likely pathogenic |  |
| *LDLR* | c.1297G>C | Missense | p.Asp433His | 8 | PM1/PM2/PP3/PP5 Likely pathogenic |  |
| *LDLR* | c.1328G>C | Missense | p.Trp443Ser | 4 | PM1/PM2/PP3/PP4 Likely pathogenic |  |
| *LDLR* | c.1339T>C | Missense | p.Ser447Pro | 2 | PM1/PM2/PP3/PP5 Likely pathogenic |  |
| *LDLR* | c.1340C>G | Missense | p.Ser447Cys | 6 | PM1/PM2/PP3/PP5 Likely pathogenic |  |
| *LDLR* | c.1432G>A | Missense | p.Gly478Arg | 4 | PM1/PM2/PP3/PP4 Likely pathogenic |  |
| *LDLR* | c.1466A>G | Missense | p.Tyr489Cys | 8 | PM1/PM2/PP3/PP5 Likely pathogenic |  |
| *LDLR* | c.1469G>A | Nonsense | p.Trp490Ter | 1 | PVS1/PM2/PM4/PP4 Pathogenic |  |
| *LDLR* | c.1474G>A | Missense | p.Asp492Asn | 4 | PM1/PM2/PP3/PP4 Likely pathogenic |  |
| *LDLR* | c.1502C>T | Missense | p.Ala501Val | 5 | PM1/PM2/PP3/PP4 Likely pathogenic |  |
| *LDLR* | c.1567G>A | Missense | p.Val523Met | 3 | PM1/PM2/PP3/PP5 Likely pathogenic |  |
| *LDLR* | c.1573G>T | Missense | p.Asp525Tyr | 2 | PM1/PM2/PP3/PP4 Likely pathogenic |  |
| *LDLR* | c.1586+1G>A | Splice-cite | NA | 2 | PVS1/PM2/PM4/PP4 Pathogenic |  |
| *LDLR* | c.1652_1662delACATCTACTCG | Frameshift | p.Asp551AlafsTer4 | 4 | PVS1/PM2/PM4/PP4 Pathogenic |  |
| *LDLR* | c.1702C>G | Missense | p.Leu568Val | 6 | PM1/PM2/PP3/PP5 Likely pathogenic |  |
| *LDLR* | c.1705+1G>C | Splice-cite | NA | 4 | PVS1/PM2/PM4/PP4 Pathogenic |  |
| *LDLR* | c.1727A>G | Missense | p.Tyr576Cys | 6 | PM1/PM2/PP3/PP4 Likely pathogenic |  |
| *LDLR* | c.1731G>T | Missense | p.Trp577Cys | 6 | PM1/PM2/PP3/PP4 Likely pathogenic |  |
| *LDLR* | c.1778dupG | Frameshift | p.Asn594GlnfsTer9 | 5 | PVS1/PM2/PM4/PP4 Pathogenic |  |
| *LDLR* | c.1783C>T | Missense | p.Arg595Trp | 4 | PM1/PM2/PP3/PP4 Likely pathogenic |  |
| *LDLR* | c.1845+2T>C | Splice-cite | NA | 23 | PVS1/PM2/PM4/PP4 Pathogenic |  |
| *LDLR* | c.1859G>C | Missense | p.Trp620Ser | 6 | PM1/PM2/PP3/PP4 Likely pathogenic |  |
| *LDLR* | c.1868T>A | Missense | p.Ile623Asn | 5 | PM1/PM2/PP3/PP4 Likely pathogenic |  |
| *LDLR* | c.1897C>T | Missense | p.Arg633Cys | 4 | PM1/PM2/PP3/PP4 Likely pathogenic |  |
| *LDLR* | c.1925T>C | Missense | p.Leu642Ser | 5 | PM1/PM2/PP3/PP4/PP5 Likely pathogenic |  |
| *LDLR* | c.1998G>C | Missense | p.Trp666Cys | 4 | PM1/PM2/PP3/PP4 Likely pathogenic |  |
| *LDLR* | c.2054C>T | Missense | p.Pro685Leu | 45 | PM1/PM2/PP3/PP4/PP5 Likely pathogenic |  |
| *LDLR* | c.2096C>T | Missense | p.Pro699Leu | 4 | PM1/PM2/PP3/PP4 Likely pathogenic |  |
| *LDLR* | c.2389G>A | Missense | p.Val797Met | 13 | PM1/PM2/PP3/PP4 Likely pathogenic |  |
| *LDLR* | c.2390-4_2393delACAGTGCT | Splice-cite | NA | 3 | PVS1/PM2/PM4/PP4 Pathogenic |  |
| *LDLR* | c.2416delG | Frameshift | p.Val806SerfsTer123 | 4 | PVS1/PM2/PM4/PP4 Pathogenic |  |
| *LDLR* | c.2416dupG | Frameshift | p.Val806GlyfsTer11 | 4 | PVS1/PM2/PM4/PP4 Pathogenic |  |
| *LDLR* | c.2431A>T | Nonsense | p.Lys811Ter | 201 | PVS1/PM2/PM4/PP4 Pathogenic |  |
| *LDLR* | c.2500G>A | Missense | p.Asp834Asn | 4 | PVS1/PM2/PM4/PP4 Pathogenic |  |
| *LDLR* | c.2579C>T | Missense | p.Ala860Val | 4 | PVS1/PM2/PM4/PP4 Pathogenic |  |
| *LDLR* | c.313-?_2311+?del | Large deletion | Truncated protein | 13 | PVS1/PM2/PM4/PP4 Pathogenic |  |
| *LDLR* | c.1186-?_1587+?dup | Large duplication | Truncated protein | 11 | PVS1/PM2/PM4/PP4 Pathogenic |  |
| *LDLR* | c.1845-?_2141+?del | Large deletion | Truncated protein | 7 | PVS1/PM2/PM4/PP4 Pathogenic |  |
| *LDLR* | c.2141-?_2311+?del | Large deletion | Truncated protein | 14 | PVS1/PM2/PM4/PP4 Pathogenic |  |
| *PCSK9* | c.94G>A | Missense | p.Glu32Lys | 42 | PS1/PS3/PP3/PP4/PP5 Pathogenic |  |

*LDLR*, LDL receptor; *PCSK9*, proprotein convertase subtilisin/kexin type 9; ACMG, American College of Medical Genetics

**Supplemental Table 2. Characteristics included and excluded in this study**

| Variables | Included | Excluded (lost to follow-up) | *P*-value |
| --- | --- | --- | --- |
|  | (N = 961) | (N = 508) |  |
| Age (years) | 52 ± 16 | 39 ± 16 | < 2.2 × 10^-16^ |
| Male (%) | 449 (46.7 %) | 228 (44.9 %) | 0.537 |
| Body mass index (kg/m^2^) | 23.0 ± 0.6 | 22.8 ± 0.6 | 0.246 |
| Hypertension (%) | 276 (28.7 %) | 21 (4.1 %) | < 2.2 × 10^-16^ |
| Diabetes (%) | 83 (8.6 %) | 20 (3.9 %) | 0.00053 |
| Total cholesterol (mg/dL) | 319 [287 – 360] | 303 [265 – 352] | 3.4 × 10^-8^ |
| Triglyceride (mg/dL) | 125 [84 – 173] | 122 [70 – 160] | 0.0042 |
| HDL cholesterol (mg/dL) | 47 [40 – 57] | 48 [40 – 58] | 0.19 |
| LDL cholesterol (at baseline, mg/dL) | 234 [206 – 279] | 221 [197 – 268] | < 2.2 × 10^-16^ |
| History of prior CVD (%) | 294 (30.6 %) | 44 (8.7 %) | < 2.2 × 10^-16^ |
| Lifestyle characteristics |  |  |  |
| Healthy dietary pattern | 740 (77.0 %) | 302 (59.4 %) | 2.8 × 10^-12^ |
| Regular exercise | 451 (46.9 %) | 236 (46.5 %) | 0.91 |
| Not smoking | 660 (68.7 %) | 388 (76.4 %) | 0.0023 |
| Absence of obesity | 864 (89.9 %) | 459 (90.4 %) | 0.856 |
| Lifestyle score | 2.8 ± 0.9 | 2.6 ± 1.0 | 0.052 |
| Favorable | 243 (25.3 %) | 94 (18.5 %) | 0.0168 |
| Intermediate | 374 (38.9 %) | 225 (44.3 %) | 0.053 |
| Unfavorable | 344 (35.8 %) | 189 (37.2 %) | 0.633 |
